# Inter-chromosomal k-mer distances

**DOI:** 10.1186/s12864-021-07952-0

**Published:** 2021-09-06

**Authors:** Alon Kafri, Benny Chor, David Horn

**Affiliations:** 1 Blavatnik School of Computer Science, Tel Aviv University, 69978 Tel Aviv, Israel; 2 School of Physics and Astronomy, Tel Aviv University, 69978 Tel Aviv, Israel

**Keywords:** Inversion symmetry, K-mer distances. Synteny

## Abstract

**Background:**

Inversion Symmetry is a generalization of the second Chargaff rule, stating that the count of a string of k nucleotides on a single chromosomal strand equals the count of its inverse (reverse-complement) k-mer. It holds for many species, both eukaryotes and prokaryotes, for ranges of k which may vary from 7 to 10 as chromosomal lengths vary from 2Mbp to 200 Mbp. Building on this formalism we introduce the concept of k-mer distances between chromosomes. We formulate two k-mer distance measures, D_1_ and D_2_, which depend on k. D_1_ takes into account all k-mers (for a single k) appearing on single strands of the two compared chromosomes, whereas D_2_ takes into account both strands of each chromosome. Both measures reflect dissimilarities in global chromosomal structures.

**Results:**

After defining the various distance measures and summarizing their properties, we also define proximities that rely on the existence of synteny blocks between chromosomes of different bacterial strains. Comparing pairs of strains of bacteria, we find negative correlations between synteny proximities and k-mer distances, thus establishing the meaning of the latter as measures of evolutionary distances among bacterial strains. The synteny measures we use are appropriate for closely related bacterial strains, where considerable sections of chromosomes demonstrate high direct or reversed equality. These measures are not appropriate for comparing different bacteria or eukaryotes.

K-mer structural distances can be defined for all species. Because of the arbitrariness of strand choices, we employ only the D_2_ measure when comparing chromosomes of different species. The results for comparisons of various eukaryotes display interesting behavior which is partially consistent with conventional understanding of evolutionary genomics. In particular, we define ratios of minimal k-mer distances (KDR) between unmasked and masked chromosomes of two species, which correlate with both short and long evolutionary scales.

**Conclusions:**

k-mer distances reflect dissimilarities among global chromosomal structures. They carry information which aggregates all mutations. As such they can complement traditional evolution studies , which mainly concentrate on coding regions.

**Supplementary Information:**

The online version contains supplementary material available at 10.1186/s12864-021-07952-0.

## Background

The phenomenon of Inversion Symmetry (IS) has recently been reevaluated and established in [1]. This generalization of the second Chargaff rule [2] implies that the number of occurrences of any sequence *m* of length k on a chromosomal strand *S* is equal to the number of occurrences of its inverse (reverse-complement) sequence *m*^*inv*^ on the same strand. Another way of stating the same fact is that the number of occurrences of *m* on one chromosomal strand is equal to the number of occurrences of *m* on the other strand provided both are being read along their own 5′ to 3′ directions.

The accuracy of such statements depends on the length k of the nucleotide sequences which are being employed. It turns out to have a monotonic dependence on k, i.e. as k increases the symmetry worsens. If one sets the required accuracy at 10% one finds [1] that it holds for k ≤ KL where KL grows logarithmically with the length L of the chromosome. KL values for mammals are 9 or 10, while for bacteria they are 7 or 8. These choices of KL guarantee that all possible k-mers of a particular k-value will be found on the chromosome in question.

Inversion symmetry can be restated as the demonstration of a low *k-mer distance* between the two strands of the same chromosome [3], with exact symmetry implying zero distance. The notion of *k-mer distances* between different chromosomes, within and between species, is a simple extension of the same basic idea: comparing frequencies of all strings of nucleotides of the same length k on different chromosomes, summing over one or over both strands of each chromosome.

Short k-mer distances can be interpreted as large structural similarities between chromosomes. In bacteria we establish correlations of short k-mer distances between bacterial strains with large synteny proximities. Both concepts are explained in the Methods section. For bacterial strains, they also serve as good measures of evolutionary distances.

The synteny proximities which we employ are valid measures between bacterial strains which are very close evolutionary relatives. Otherwise one cannot find large genomic sections with high identities among them. Therefore, conventional synteny measures which are used in genomic evolutionary studies [4] are very different from our synteny proximities and are mostly concentrated on coding regions.

k-mer distances, which are global measures, can be used to compare any two chromosomes. When studying eukaryotes, the compared chromosomes are dominated by non-coding regions. Comparing minimal k-mer distances between various genomes, we find interesting results. In particular, ratios of unmasked to masked minimal genome distances, correlate with evolutionary distances among different species.

## Methods

### Definitions and properties of k-mer distances between chromosomes

The term *k-mer* refers (in the genomic context) to all possible nucleotide substrings of length k that are contained in a given chromosomal strand of length L, uncovered by a sliding-window search. The total number of their occurrences is N = L-k + 1. We define the empirical frequency of a specific k-mer, e.g. *m*_*1*_, in the strand S as the number of occurrences of this k-mer in S divided by N

1 $$ {f}_{m_1}=\frac{n\left({m}_1\right)}{N} $$

Let us define the k-mer distance D_1_ as the L1-norm of the difference between k-dim vectors containing frequencies of all k-mers, when comparing two chromosomal strands (e.g. positive strands of two chromosomes) S_1_ and S_2_:

2 $$ {D}_1^k\left({S}_1,{S}_2\right)={\sum}_{i=1}^{4^k}\mid {f}_{m_i}\left({S}_1\right)-{f}_{m_i}\left({S}_2\right)\mid $$

The index 1 in D_1_ refers to the fact that we use only one strand on each chromosome in this comparison of two chromosomes.

Similarly, we may define a distance measure D_2_ by taking into account both strands of the two chromosomes, reading them along their own 5′ to 3′ directions. Since each specific k-mer on the negative strand, is accompanied by its inverse (reverse-complement) on the positive strand, we may define D_2_ as

3 $$ {D}_2^k\left({S}_1,{S}_2\right)={\sum}_{i=1}^{4^k}\left|{f}_{m_i}\left({S}_1\right)+{f}_{M_i}\left({S}_1\right)-{f}_{m_i}\left({S}_2\right)-{f}_{M_i}\left({S}_2\right)\right|/2 $$

where we use a single strand on each chromosome and define for every k-mer its inverse (reverse complement)

$$ {M}_i={m}_i^{inv} $$

and sum over all of them along a single strand of each of the two chromosomes. Division by 2 is introduced in the definition of D_2_ because the effective number of counts on each chromosome becomes 2 N.

The triangular inequality implies that

4 $$ \mid {f}_{m_i}\left({S}_1\right)+{f}_{M_i}\left({S}_1\right)-{f}_{m_i}\left({S}_2\right)-{f}_{M_i}\left({S}_2\right)\left|\le |{f}_{m_i}\left({S}_1\right)-{f}_{m_i}\left({S}_2\right)\right|+\left|{f}_{M_i}\left({S}_1\right)-{f}_{M_i}\left({S}_2\right)\right| $$

for every single k-mer. It follows then that

5 $$ {D}_2^k\left({S}_1,{S}_2\right)\le {D}_1^k\left({S}_1,{S}_2\right) $$

Using the above definitions we summarize the properties of k-mer distances:

1. Positivity. By definition all distances are non-negative.
2. If ${D}_{1,2}^k\left({S}_1,{S}_2\right)=0$ then *S*_*1*_ and *S*_*2*_ are equivalent, in the sense that both chromosomes have the same frequencies of all k-mers. This does not necessarily imply that the two chromosomes are equal to each other, because they may differ in length.
3. Symmetry. By definition, ${D}_{1,2}^k\left({S}_1,{S}_2\right)={D}_{1,2}^k\left({S}_2,{S}_1\right)$.
4. Inequality: ${D}_2^k\left({S}_1,{S}_2\right)\le {D}_1^k\left({S}_1,{S}_2\right)$, as proved above in Eq. 5.
5. Triangular inequalities of distances:

6 $$ {D}_{1,2}^k\left({S}_1,{S}_3\right)\le {D}_{1,2}^k\left({S}_1,{S}_2\right)+{D}_{1,2}^k\left({S}_2,{S}_3\right). $$

This can be proved in an analogous fashion to property 4.

6. Inversion symmetry [1] implies that ${D}_1^k\left({S}_1,{S}_2\right)=0$ if *S*_*2*_ is the inverse of *S*_*1*_ (or equivalent to it in the sense of property 2). Otherwise this distance will be positive. Such a definition of inversion symmetry has been introduced by [3]. ${D}_2^k\left({S}_1,{S}_2\right)=0$ is a trivial statement for two strands which are inverses of each other.
7. Monotonic increase with k:

7 $$ {D}_{1,2}^{k-1}\left({S}_1,{S}_2\right)\le {D}_{1,2}^k\left({S}_1,{S}_2\right) $$

To prove this property note that a k-mer m_i_^k^ can be generated from a corresponding m_j_^k-1^, which coincides with all first k-1 entries of m_i_^k^, by adding to it one of the four nucleotides {A, C, G, T}. Let us define this set as {j,i} for a given m_j_^k-1^ and four corresponding m_i_^k^. It follows then that

$$ {\displaystyle \begin{array}{c}{D}_1^{k-1}\left({S}_1,{S}_2\right)={\sum}_{j=1}^{4^{k-1}}\mid {f}_{m_j}\left({S}_1\right)-{f}_{m_j}\left({S}_2\right)\mid \le \\ {}{\sum}_{i=1}^{4^k}\mid {f}_{m_i}\left({S}_1\right)-{f}_{m_i}\left({S}_2\right)\mid ={D}_1^k\left({S}_1,{S}_2\right)\end{array}} $$

by summing over the indices using the {j,i} association, and applying the extended triangular inequality to each set of four f_i_ whose k-mers m_i_^k^ begin with the same (k-1)-mer m_j_^k-1^ with index j.

This proof can be trivially extended to D_2_.

One condition for these inequalities to hold is that all k-mers are realized on the chromosomal strands which are being investigated, i.e. all $n\left({m}_i^k\right)>0$.

Finally we touch upon the question of the range of k-values for which the distance measures can be applied.

Shporer et al. [1] have introduced the notion of the KL limit. This is the k-value for which Inversion Symmetry fails at the rate of 10%. They demonstrated that chromosomes of different species, as well as different human chromosomal sections, follow a universal logarithmic slope of KL ~ 0.7 ln(L), where L is the length of the chromosome. This limit can also be derived from the assumption that L> > 4^k^ allowing for all k-mers to be expressed on the chromosome.

**Fig. 1.**
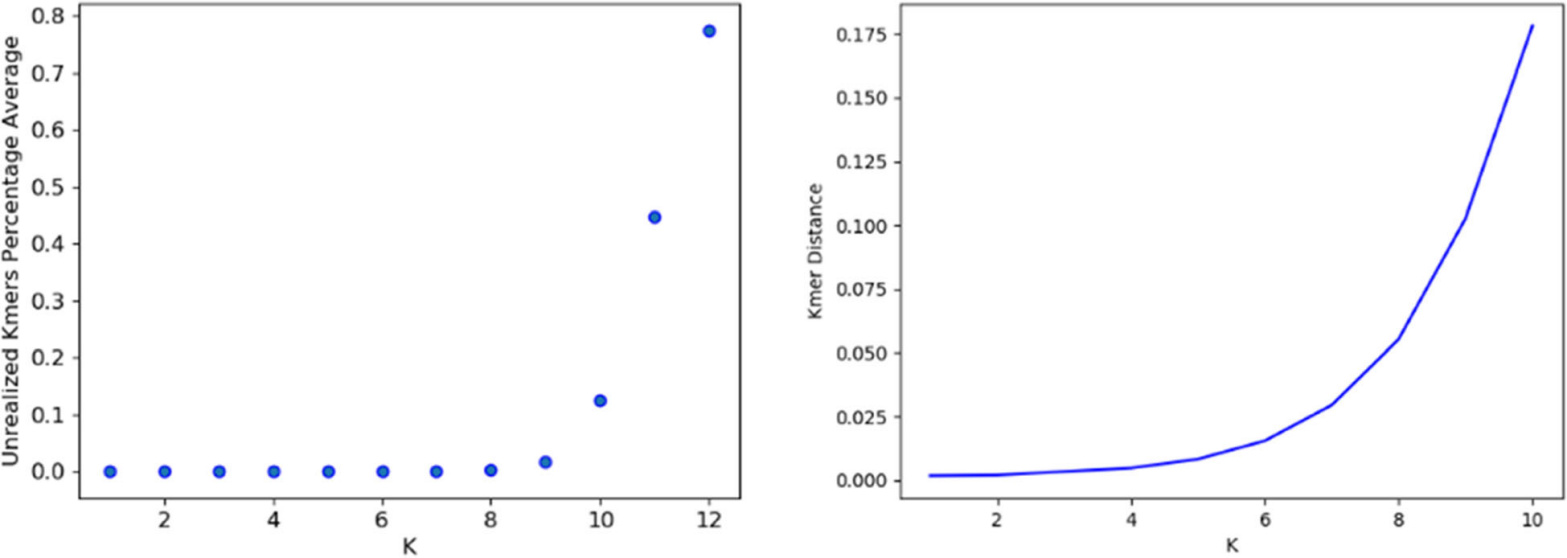
k-mer analysis of *E. coli*, for which KL = 7. **a** Percentage of missing k-mers, i.e., those for which $n\left({m}_i^k\right)=0$. **b** D_1_ distance between two K12 strains of *E. coli*

As an example of relevant statistics we display in Fig. 1 the percentage of missing k-mers, i.e. those which do not appear on the strand, and the distance between two close strains of *E. coli* as function of k, demonstrating that good results are obtained for k ≤ KL = 7.

When evaluating distances between two chromosomal strands with different lengths, L_1_ and L_2_, one should limit oneself to KL where L = min(L_1_, L_2_), guaranteeing that the same k is valid for both chromosomal strands which are being compared.

We provide a python program for calculating k-mer distances between two chromosomes, given as fasta files, in (https://github.com/akafri/k-mer-distances).

### Definition of synteny distances

**Fig. 2:**
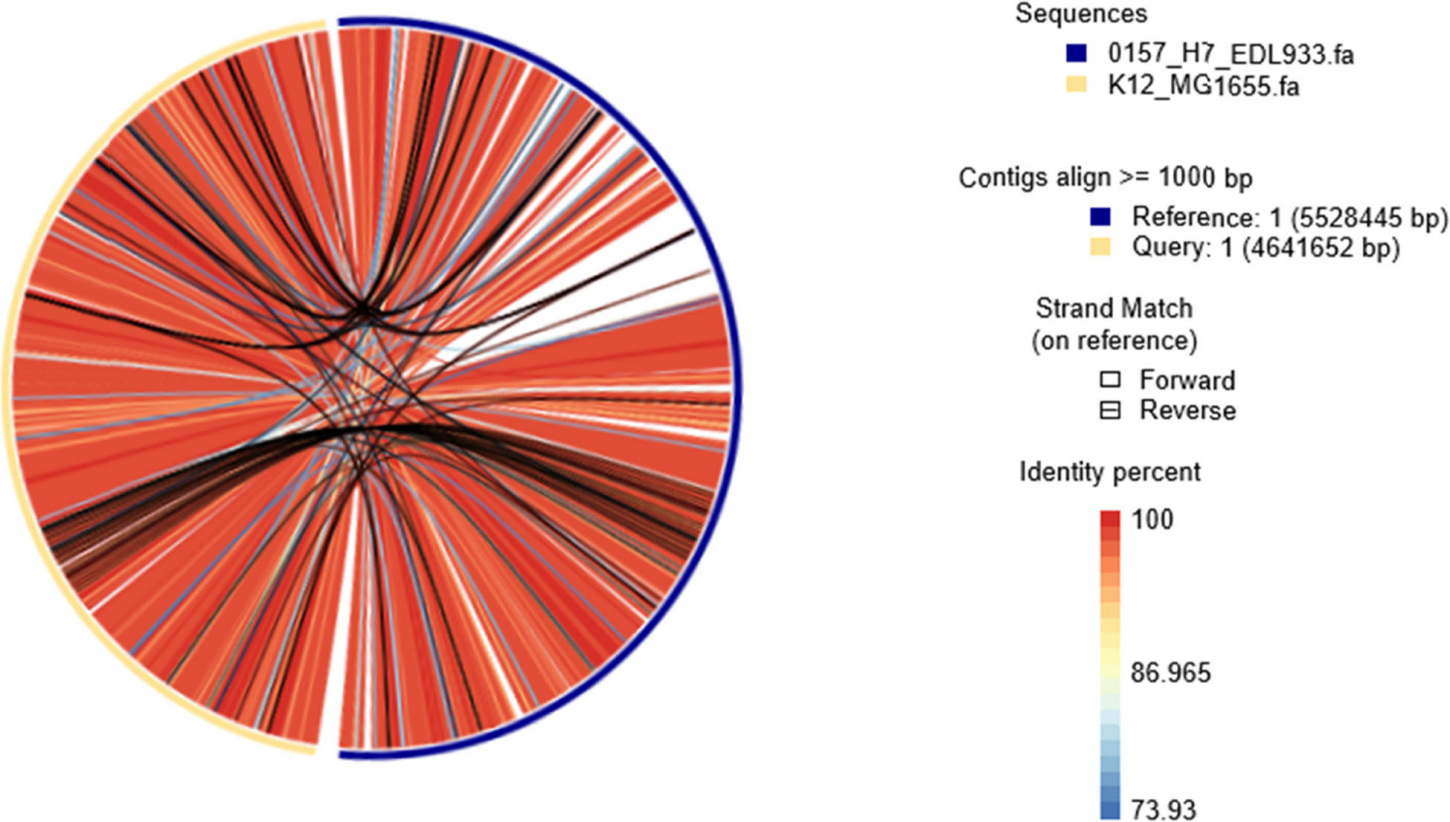
Synteny Blocks between *E. coli* 0157-H7-EDL933 (right) and *E. coli* K12-MG1655 (left). The colors represent the Identity Percentage where red indicates high identity percentage of DSB and blue indicates low identity percentage of DSB. The black colors represent ISBs

Synteny blocks are genetic sequences in genomes of two species which consist of aligned homologous genes. A recent example of their importance was demonstrated by [5, 6]. Here we introduce definitions of synteny distances, which will be used to compare with k-mer distances. This comparison will be carried out using different strains of the same bacterium, where large synteny blocks with identity percentages higher than 90% exist. The threshold of 90% is arbitrary. It was made to guarantee high similarity between the relevant chromosomes. For bacteria, where the selection of a positive strand is well defined, we differentiate between Direct Synteny Blocks (DSB), appearing along the same strand in both genomes, and Inverse Synteny Blocks (ISB), lying on opposite strands. An example is shown in Fig. 2.

Searching for synteny blocks, BLAST was first used to identify local alignments between the full two sequences. The R package OmicCircus [7] was used to visualize results. From the BLAST output, we extract synteny blocks that have identity percentages higher than 90%, and calculate the overall sequence lengths of DSB and ISB (L_DSB_ and L_ISB_) respectively.

We then define direct synteny proximity

8 $$ {P}_{DSYN}\left({S}_1,{S}_2\right)=\frac{L_{DSB}}{\min \left({L}_1,{L}_2\right)}, $$

and overall synteny proximity as

9 $$ {P}_{SYN}\left({S}_1,{S}_2\right)=\frac{L_{DSB}+{L}_{ISB}}{\min \left({L}_1,{L}_2\right)} $$

where L_1_ and L_2_ are the lengths of the chromosomes S_1_ and S_2_ which are being compared.

### The matched-pair algorithm for k-mer distances between two species

To define distances between two eukaryote genomes we started by evaluating a distance matrix between all chromosomes of the two species. We then constructed a graph whose vertices are the chromosomes of the two species and its edges (lines connecting the vertices) represent the distance value of each pair. We proceeded along the following algorithmic steps:

1. Eliminate edges with distances > 1 from the graph.
2. Define an empty distance vector.
3. Find the edge of the graph with the lowest distance value.
4. Add this value as an entry to the distance vector.
5. Remove this edge from the graph and repeat from step 3 until the graph is exhausted.
6. Inspect the resulting distance vector and report its minimum (the first edge considered by the matching algorithm) and its median.

## Results

### Distance measures in bacteria

We compared genomes of 23 strains of *E. coli* and 14 strains of *Salmonella enterica*. They are listed in Tables 1 and 2.

**Table 1:** *E. coli* data, taken from [8]. See also data supplementary file

Id	Organism	Size (bp)	No. genes	Accession Number
1	*E. coli 0157:H7 EDL933*	5,620,522	5312	AE005174
2	*E. coli 0157:H7 Sakai*	5,594,477	5230	BA000007
3	*E. coli 0111:H- 11128*	5,766,081	5407	AP010960
4	*E. coli O26:H11 11,368*	5,851,458	5516	AP010958
5	*E. coli 536*	4,938,920	4620	CP000247
6	*E. coli 55,989*	5,154,862	4763	CU928145
7	*E. coli APECO1*	5,497,653	4428	CP000468
8	*E. coli CFT073*	5,231,428	5339	AE014075
9	*E. coli 0127:H6 E2348/69*	5,069,678	4554	FM180568
10	*E. coli E24377A*	5,249,288	4749	CP000800
11	*E. coli 0157:H7 EC4115*	5,704,171	5315	CP001164
12	*E. coli ED1a*	5,209,548	4915	CU928162
13	*E. coli HS*	4,643,538	4378	CP000802
14	*E. coli IAI1*	4,700,560	4353	CU928160
15	*E. coli K12 MG1655*	4,639,675	4149	U00096
16	*E. coli K12 W3110*	4,646,332	4226	AP009048
17	*E. coli B str. REL606*	4,629,812	4205	CP000819
18	*E. coli S88*	5,032,268	4696	CU928161
19	*E. coli SE11*	5,155,626	4679	AP009240
20	*E. coli SE15*	4,839,683	4488	AP009378
21	*E. coli SMS-3-5*	5,215,377	4743	AP009378
22	*E. coli UMN026*	5,324,391	4826	CU928163
23	*E. coli UTI89*	5,179,971	5021	CP000243

**Table 2:** *Salmonella enterica* data. Taken from NCBI [9]. See also data supplementary file

Id	Organism	Size (bp)	Accession Number
1	*S. enterica serovar Typhimurium*	4,951,383	ASM694v2
2	*S. enterica serovar Typhi*	5,133,713	ASM19599v1
3	*S. enterica serovar Choleraesuis*	4,944,000	ASM810v1
4	*S. enterica serovar Enteritidis*	4,685,848	ASM950v1
5	*S. enterica serovar Gallinarum*	4,658,697	ASM952v1
6	*S. enterica serovar Paratyphi A*	4,585,229	ASM1188v1
7	*S. enterica serovar Newport*	5,007,719	ASM1604v1
8	*S. enterica serovar Paratyphi C*	4,888,494	ASM1838v1
9	*S. enterica serovar Paratyphi B*	4,858,887	ASM1870v1
10	*S. enterica serovar Heidelberg*	4,983,515	ASM2070v1
11	*S. enterica serovar Schwarzengrund*	4,823,887	ASM2074v1
12	*S. enterica serovar Agona*	4,836,638	ASM2088v1
13	*S. enterica serovar Dublin*	4,917,459	ASM2092v1
14	*S. enterica serovar Montevideo*	4,694,375	ASM18895v5

**Fig. 3:**
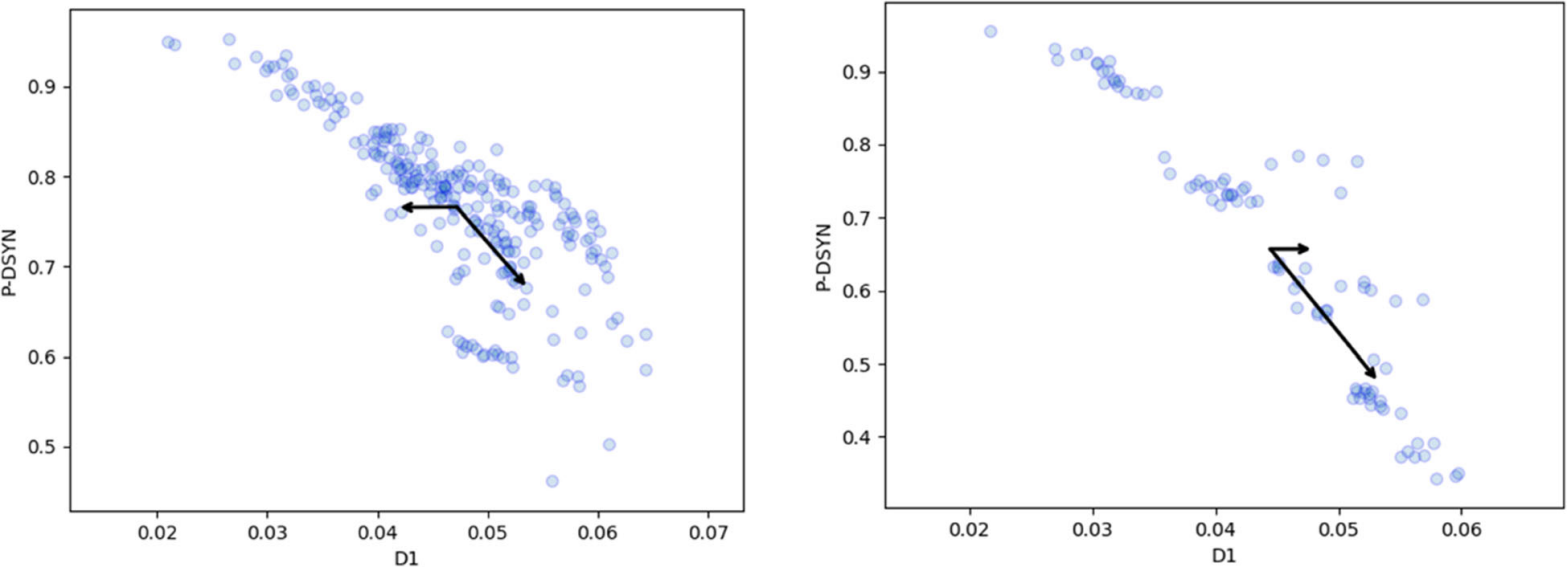
Comparison of P_DSYN_ with D_1_^k = 7^ for pairs of (left) *E. coli* strains and (right) *S. enterica* strains. Each dot represents a pair of strains. Arrows indicate the two principal components of PCA applied to the data points in the diagram, delineating the variance of the data along these two directions

In Fig. 3 we present correlations of P_DSYN_ with D_1_ for (a) *E. Coli* and for (b) *S. enterica* strains. In each of the two data sets we have looked into all pairs of strains. The data are presented for k = 7. We report only results between strains of the same bacterium since no significant correlation was found between any two strains of the two different bacteria. The higher statistics of *E. coli* leads to a clearer observation of the correlations.

**Fig. 4:**
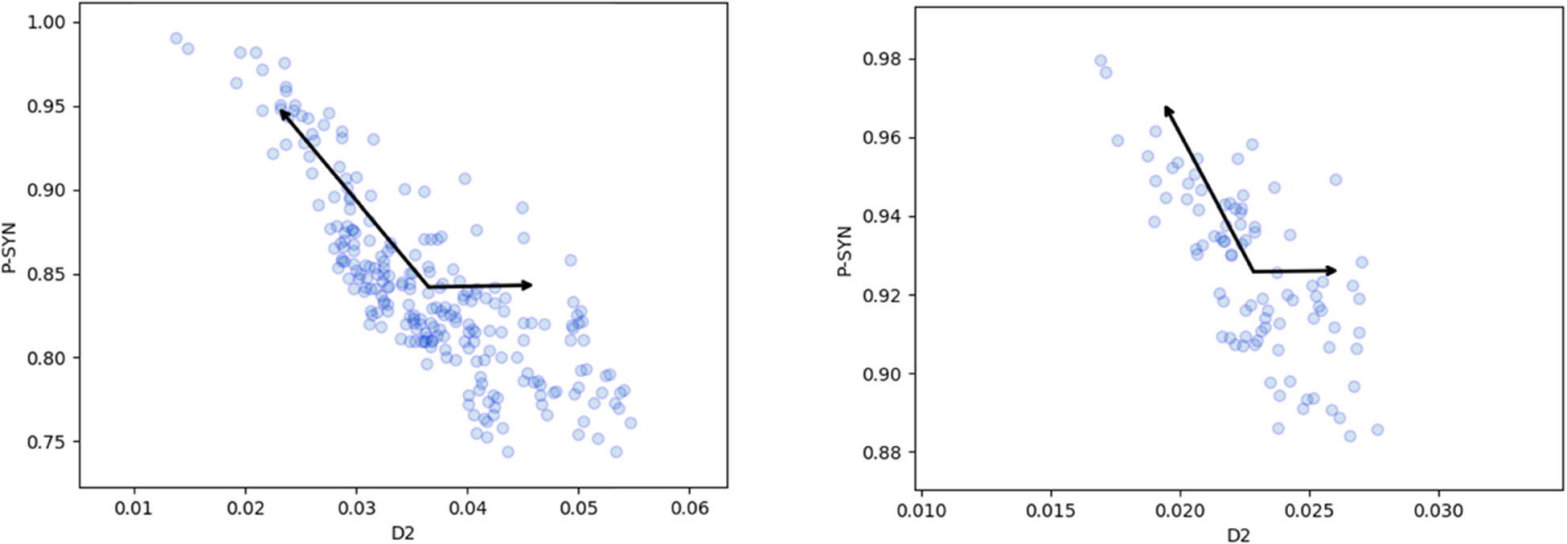
Comparison of P_SYN_ with D_2_^k = 7^ for pairs of (left) *E. coli* strains and (right) *S. enterica* strains

Next we turn to correlations of over-all synteny with D_2_^k = 7^. This is presented in Fig. 4. Once again we note the strong correlations in the data. The strong negative correlation is particularly significant for the *E. coli* strains where we have many more pairs of strains which can be compared with one another. Hence we limit our further analysis to just *E. coli* strains.

**Fig. 5:**
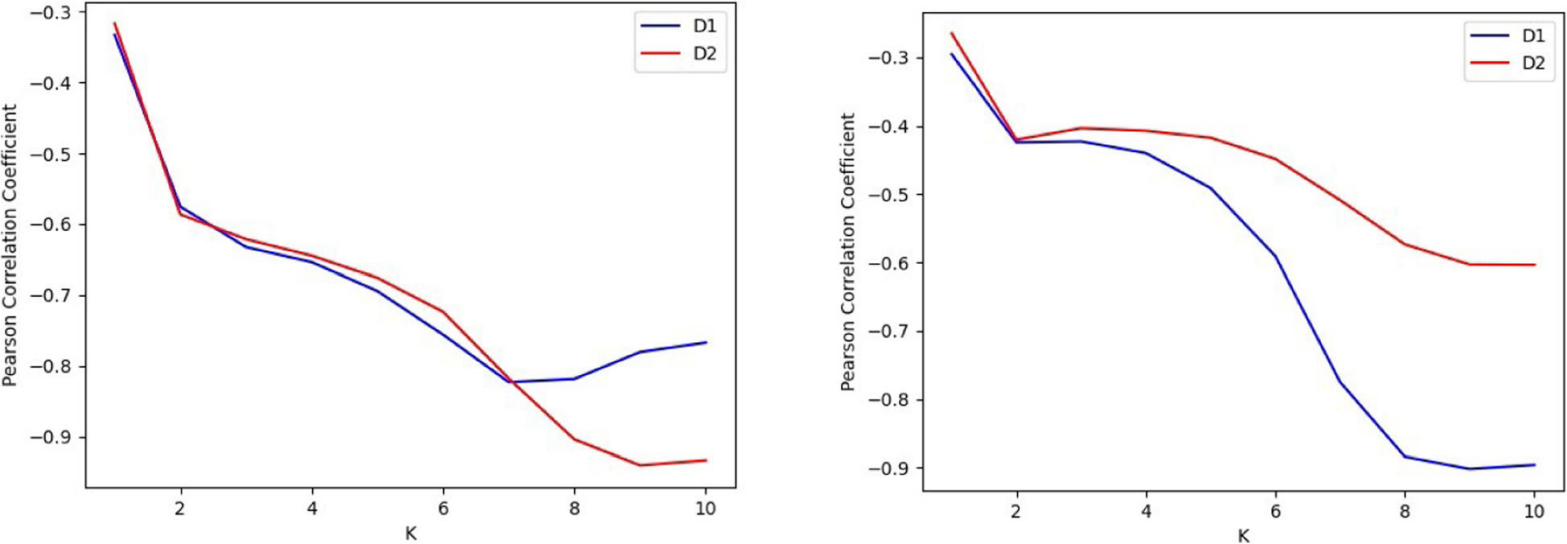
Pearson correlation coefficients of the two k-mer distance measures of pairs of *E. coli* strains as function of k, with (left) P_SYN_ and (right) P_DSYN_. We present only results of *E. coli* strains, because of the larger number of pairs of strains, which leads to higher statistical significance

In order to appreciate the variation with k we display in Fig. 5 the Pearson correlation coefficients of D_1_ and D_2_ for all *E. coli* pairs of strains, as function of k, for the two classes of synteny measures. Clearly k = 7, the choice made in Figs. 3 and 4, leads to a strong correlation, as observed in Figs. 3 and 4. The relevant Pearson correlation *p*-values turn out to be miniscule, with the highest one being of order 10^− 7^ for k = 1 for both D_1_ and D_2_, and others of order 10^− 22^ and smaller.

We find different correlations of the two measures with P_DSYN_. Whereas D_1_ displays the expected negative correlation, for all relevant k, D_2_ is less sensitive to the direct synteny measure. This may be expected since D_2_ is a measure sensitive to both strands whereas P_DSYN_ is sensitive to only one strand in each chromosome.

In order to appreciate this result let us dwell on the question why inversion symmetry [1] holds up to large k-values of order KL. The plausible explanation is that genomes evolve through rearrangement processes. These rearrangements are inversions of sections between two breakpoints on the same chromosome. They may follow one another in a nested fashion. This scenario can explain the observed inversion symmetry, as demonstrated in [1]. Pevzner and Tesler [5] have argued that such phenomena are the basis of chromosomal evolution for single chromosomes and, with lower probability, also between different chromosomes. Here we observed that D_1_ between two strains of bacteria correlates strongly with both P_DSYN_ and P_SYN_ for all k ≤ 7, both reflecting chromosomal evolution at the short evolutional scale appropriate to different strains of the same bacteria.

### Distance measures between different species

In the previous section we have analyzed k-mer distances between closely related bacterial strains, where the synteny distances that we have defined can be easily observed. When evolutionary genomics is applied to different eukaryotes one often limits oneself to similarity between homologous proteins rather than accurate duplications or inversions of large sections of the DNA. The use of k-mer distances can indicate similarities between full chromosomes, which is the study we propose. From Inversion Symmetry we learn the powerful effect of rearrangement within a single chromosome. Rearrangements may also occur between chromosomes and k-mer distances reflect their effects.

Evaluating minimal D_2_ distances according to the matched-pair algorithm (see Methods) we obtain the results displayed in Tables 3 and 4. The genome inputs, both unmasked (Table 3) and masked (Table 4), are taken from the UCSC server (see data supplementary file). Clearly, there is quite a difference between the two choices: masking reduces the distance values considerably. We use k = 8 which is a choice appropriate for all displayed species in Tables 3, 4, 5 and 6.

**Table 3:**
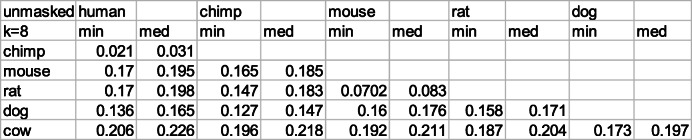
Minimal and median D_2_^k = 8^ distances between six genomes belonging to different mammals, for unmasked versions of the genomes. See Methods for definition of the computational procedure

**Table 4:**
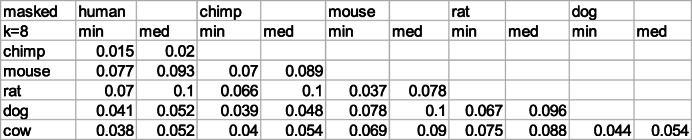
Minimal and median D_2_^k = 8^ distances between masked genomes of different mammals. See Methods for definition of the computational procedure

**Table 5:**
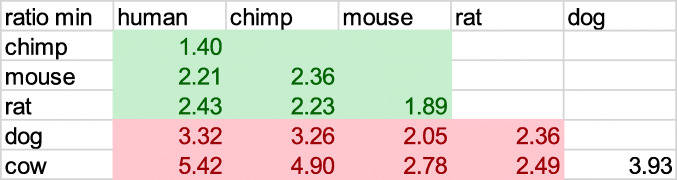
Ratio of unmasked to masked minimal D_2_^k = 8^ distances. The ratios among primates and rodents are correlated with evolutionary time estimates (http://www.timetree.org/), but this is not true for the rest of this table

**Table 6:**
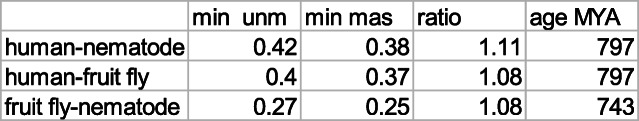
Unmasked and masked minimal D_2_^k = 8^, their ratios, defined as KDRs, and the separation age estimates derived from (http://www.timetree.org/)

There are several striking results in the two tables 3 and 4. One important result is the closeness of minimal and medial distance values. This implies that similar k-mer distances are observed for many chromosomal pairs of the two genomes, and are not limited to a single particular pair of chromosomes. In other words, homology spreads out between different chromosomal sections of the two compared species.

Another important result is the huge difference between minimal k-mer distances of unmasked and masked genomes. Conventional understanding regards the low complexity components of the unmasked regions as unprotected by evolution. Hence ratios of unmasked to masked minimal D_2_^k = 8^ distances measure the aggregated effect of different strengths of mutations when the low complexity sections of genomes are taken into account.

The results for these ratios are presented in Table 5. They seem to be correlated to evolutionary time lapses among primates and rodents, where the separation between human and chimpanzee is dated at 6.7 MYA (million years ago), between mouse and rat 20 MYA and between rodents and primates 90 MYA. However the correlation between all four to dog and cow, ceases to exist. The separation age between the primates to dog and cow is estimated at 96 MYA and between dog and cow 78 MYA. All the evolutionary estimates are derived from the time-tree website (http://www.timetree.org/).

A major tool employed in genomic evolutionary studies is Reversal (or inversal) Distance (RD) [5, 6]. Concentrating on the orders and details of genes or other markers, the idea is to work out how many inversions take place along the evolutionary path from one species to another. RD is the minimum number of reversals required to transform one genome into the other. The web-tool of (http://www.timetree.org/) can be used to evaluate such distances. They fit much better the evolutionary time estimates, which is somewhat a tautology because the estimates of (http://www.timetree.org/) take the RD methodology into account. However, RD is problematic when very large evolutionary distances are concerned, because of the shortage in genes which can be compared between distant organisms. K-mer distances are not subject to such constraints. Hence they can be applied to such problems. In Table 6 we compare human with the nematode (*C. elegans*) and the fruit fly (*D. melanogaster*), using the same methods as in Table 5. Obviously these results are satisfactory.

Interestingly, k-mer distances are immune to large inversion events. In fact, this was the reason we use them to begin with, starting with the lessons drawn from Inversion Symmetry of chromosomes. On the other hand, k-mer distances are sensitive to all other mutations that occur along an evolutionary path. In this sense, K-mer minimal Distance Ratios among genomes (KDR) can serve as a complement to RD. Moreover, it is applicable to all eukaryotes.

The full potential of KDR has still to be investigated and explained. Evolutionary genomic tools deal extensively with substitution rates, in particular the non-synonymous ones affecting amino-acid changes in proteins. The analogous investigation of substitution rates in low-complexity and high-complexity genomic regions is needed to explain how KDR, or the various minimal or median k-mer distances among genomes, can be used for meaningful evolutionary conclusions.

## Conclusions

We have introduced measures of k-mer distances, and applied them to bacteria and to eukaryotes. The two measures D_1_ and D_2_ were compared to synteny measures in bacteria, tracing large identical sections of chromosomes between two strains of the same species. We identified a strong correlation between D_1_ and direct syntenic regions and a strong correlation between D_2_ and both direct and inverse syntenies, which indicates evolutionary similarity between two strains. We argue therefore that k-mer distances are validated as good measures for evolutionary distances within bacteria.

D_2_ measures are also adequate for estimating distances between any two genomes which may have very ancient common ancestors. We exemplify this fact by demonstrating such distance measures between several eukaryotes. We find that there exists considerable difference between masked and unmasked distances, as expected from common evolutionary understanding of rapid variation in low complexity regions, being less protected by evolution. Moreover, we exploit this difference to establish minimal K-mer Distance Ratios (KDR), which correlate with evolutionary time scales of primates and rodents, as well as very large time scales such as between human, nematode and fruit fly.

Whereas conventional evolutionary studies continue to use traditional methods following changes within and throughout homologous genes, our k-mer distances take into account the full chromosomes, involving both coding and non-coding sections. As such, they carry novel information which complements traditional investigations.

## Supplementary Information

**Additional file 1.**

## Data Availability

All data analyzed during this study are included in the data supplementary information file.
