# Supplementary material for: Inter-chromosomal k-mer distances
Source: BMC Genomics. 2021 Sep 6;22:644. doi: 10.1186/s12864-021-07952-0 (PMC8422766; doi:10.1186/s12864-021-07952-0)
Supplement: Supplementary file 1 — Additional file 1. [file 12864_2021_7952_MOESM1_ESM.docx]

Data used in this study are the following:


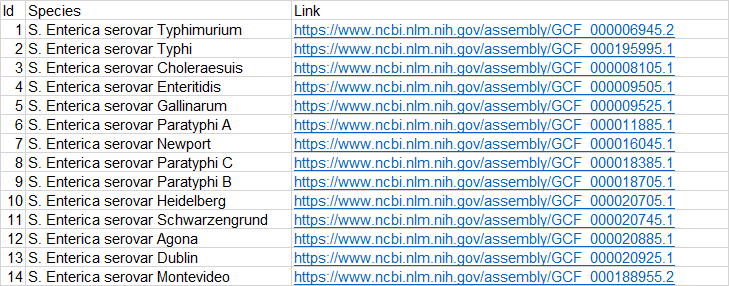

Eukaryotes:

human (hg38) - https://hgdownload.soe.ucsc.edu/goldenPath/hg38/chromosomes/

chimp (panTro5) - https://hgdownload.soe.ucsc.edu/goldenPath/panTro5/bigZips/

mouse (mm10) - https://hgdownload.soe.ucsc.edu/goldenPath/mm10/bigZips/

rat (rn6) - https://hgdownload.soe.ucsc.edu/goldenPath/rn6/bigZips/

dog (canFam3) - https://hgdownload.soe.ucsc.edu/goldenPath/canFam3/bigZips/

cow (bosTau8) - https://hgdownload.soe.ucsc.edu/goldenPath/bosTau8/bigZips/

nematode (C elegans) - <https://hgdownload.soe.ucsc.edu/goldenPath/ce11/chromosomes/>

fruit fly (D melanogaster) - https://hgdownload.soe.ucsc.edu/goldenPath/dm6/bigZips/
